# Shame and Suicidal Ideation among Undergraduates in China: The Mediating Effect of Thwarted Belongingness and Perceived Burdensomeness

**DOI:** 10.3390/ijerph17072360

**Published:** 2020-03-31

**Authors:** Jingjing Zhao, Yanna Chi, Yanli Ju, Xiyao Liu, Jingjing Wang, Xinglai Liu, Bob Lew, Ching Sin Siau, Cunxian Jia

**Affiliations:** 1 School of Marxism, Shandong University, Jinan 250012, China; 2 School of Public Health, Shandong University, Jinan 250012, China; 3 Department of Social Psychology, Putra University of Malaysia, Serdang 43400, Selangor Malaysia; 4 Faculty of Social Sciences and Liberal Arts, UCSI University, Kuala Lumpur 56000, Malaysia

**Keywords:** suicidal ideation, shame, thwarted belongingness, perceived burdensomeness, undergraduate students, China

## Abstract

Undergraduate students with shame are more likely to experience suicidal ideation, but there remains a lack of research investigating the factors underlying this relationship. The interpersonal theory of suicide posits that suicidal ideation is influenced by the simultaneous presence of thwarted belongingness and perceived burdensomeness. We examined the prevalence of suicidal ideation among undergraduate students in China and examined the association between shame and suicidal ideation mediated by perceived burdensomeness and thwarted belongingness. A survey was conducted in July 2018 involving 2320 undergraduate students, and the twelve-month prevalence of suicidal ideation was 8.95%. Shame played a crucial role in predicting suicidal ideation, and the mediating effects of perceived burdensomeness and thwarted belongingness between shame and suicidal ideation were significant. Suicidal ideation is common among undergraduate students in China and merits greater attention. Shame, perceived burdensomeness, and thwarted belongingness may be important factors to assess among undergraduate students in suicide risk assessment and psychological intervention.

## 1. Introduction

Suicide is an important global public health problem and the second leading cause of death in young people aged 15–29 years worldwide [1,2,3]. Currently, although the suicide rate in China has decreased rapidly (22.2 per 100,000 in 1993 to 6.60 per 100,000 in 2012) [4], suicide is still a significant problem that warrants serious attention [5]. Suicidal ideation, defined as thoughts of self-harming or killing oneself [6], is an important indicator in the assessment of suicide risk [7]. Thus, it is imperative to identify the characteristics and related factors of suicidal ideation and to develop effective prevention and intervention measures that reduce suicidal ideation.

College students, a majority of whom are within the 17–25-year-old age bracket, could be affected by adaptation to college life, self-identity, interpersonal relationships, and career development. In the worldwide, they are a specific group who report high levels of suicidal ideation, planning, and attempts [8,9]. Data indicate that the suicide rate of college students is 2–4 times higher than the rate of noncollege students of the same age [10]. In China, suicide is the second leading cause of death among college students, following accidental injury [11]. Most college students were born post-1990s when the “One-Child” policy was mandatory, which means he or she is the only child in his or her family. The upbringing as an only child has been argued as leading to their relative impulsiveness and inability to withstand negative life events, compared with young adults with siblings [12]. A meta-analysis showed that the overall pooled prevalence of suicidal ideation was approximately 10.72% among college students in China [12], which was higher than the general population (3.9%) [13]. Therefore, suicidal ideation in Chinese college students merits greater attention and should be further examined.

Shame is a painful self-conscious emotion that may be triggered by others’ negative evaluation, and can bring about a debilitating experience and a strong denial of oneself [14,15,16]. It is a complex multidimensional experience that can involve negative cognitive beliefs about oneself, feelings such as anxiety and disgust, behavioral tendencies such as withdrawal or avoidance, and a range of adverse psychological effects [17,18].

In the past 20 years, several instruments have been developed to assess shame, such as the Personal Feelings Questionnaire [19], Experience of Shame Scale [20], the Test of Self-conscious Affect–3 [21], Guilt and Shame Proneness Scale [22], Multidimensional Shame-Related Response Inventory–21 [15]. Shame can serve the adaptive or maladaptive function in maintaining mental health and social functioning, which depends on an individual’s response to shame [23]. In maladaptive outcomes of shame, it causes avoidance and withdrawal from society and impairs social functioning. A meta-analysis on the communication of suicidal intentions showed that nearly half of those who went on to suicide had communicated about killing themselves, which could be a sign of help-seeking [24]. However, those who have high levels of shame may be less likely to communicate their suicidal intention with others, or they could have tried to communicate using indirect means but had failed to be understood. Besides, many studies have shown that shame is one of the factors leading to suicidal ideation. Studies among college students and the general population revealed that shame was positively associated with suicidal ideation [25,26,27]. Furthermore, among veterans, the experience of shame mediates the relationship between post-traumatic stress disorder symptoms and suicidal ideation [28].

There is much evidence that shame predicts suicidal ideation [28,29,30], but there remains a relative lack of research investigating the factors underlying this relationship. Drawing from theoretical suicide models may be helpful in understanding this relationship. The interpersonal theory of suicide [31], one of the most widely studied suicide theories, posits that suicidal ideation is associated with the simultaneous presence of thwarted belongingness and perceived burdensomeness. Thwarted belongingness is the feeling that one is alienated from others and that he or she cannot be a part of a family, a circle of friends, or another valued group [32]. Perceived burdensomeness includes the sense that one is a burden to his or her family, friends, and/or society [32]. The theory also suggests that thwarted belongingness and perceived burdensomeness are proximal psychological states of suicidal ideation [28]. Over the past decade, considerable support of the theory has emerged across various populations, including adult outpatients [33,34], young adolescents [35], college students [36], deployed military personnel [37], and older adults [28,38,39].

From the perspective of the interpersonal theory of suicide, shame may lead to suicidal ideation by increasing or exacerbating perceived burdensomeness and thwarted belongingness. Brown et al. noted that experiencing shame evoked a sense of being damaged and undeserving of love and belonging [40]. Shame creates pervasive feelings of fear, blame, disconnection, and unworthiness, which damages relationships because the shamed individual attempts to hide, blame, and withdraw from others [23,41]. It is supposed that individuals may blame themselves more readily for perceived failures and rejections in social situations when experiencing shame [42]. Thereafter, their response to shame could be self-devaluation, fear of social consequences, and adoption of maladaptive behavioral tendencies. They may feel like a burden to others. In addition, shame may diminish one’s reliance on friends and family and cause individuals to withdraw from and avoid social contact [3], which may lead to a loss of perceived belongingness. Trauma-related shame was found to have a partially mediating effect on sexual assault severity and perceived burdensomeness/thwarted belongingness [43]. Furthermore, a study on Chinese-American breast cancer survivors suggested that perceived burdensomeness could be a mediator of the association between self-stigma and quality of life [44]. It is noted that the conception of self-stigma in the study is synonymous with shame.

Based on these findings, we hypothesize that thwarted belongingness and perceived burdensomeness may mediate the relationship between shame and suicidal ideation. The specific objectives of the present study are as follows: (1) to estimate the prevalence of suicidal ideation among college students; (2) to investigate whether shame, thwarted belongingness, and perceived burdensomeness could significantly predict suicidal ideation; and (3) to explore the mediating effects of thwarted belongingness and perceived burdensomeness between shame and suicidal ideation.

## 2. Materials and Methods

### 2.1. Participants and Data Collection Procedure

This cross-sectional study was conducted among 2320 undergraduate students in a university in Jinan, Shandong Province, China, in July 2018. We selected eight colleges as the primary sampling unit from the university. All classes in each college were numbered, and then 1–2 classes were randomly selected in each grade. All the students in the selected class were included in the study. Before the survey, researchers explained to the participants the purpose of the research and the specific requirements of completing the questionnaire, such as option types and the instructions for the scales. Participation was voluntary and the respondents were informed to fill in the questionnaire after reading the participant information sheet and providing written informed consent. All the participants filled in the questionnaire during the break time between classes. They took approximately 20 min to complete it. A total of 2320 questionnaires were given out, of which 241 were considered invalid because of missing data. A total of 2079 valid questionnaires were used in this study. The response rate was 89.61%.

### 2.2. Measures

#### 2.2.1. Social Demographic Variables

The demographic information collected included individual and household data. Individual-level variables included age, gender, grade, major, and ethnicity. Majors included liberal arts, sciences, engineering, and medicine. Ethnicity was categorized as Han ethnicity or ethnic minorities. The variables at the household level included whether the participant was an only child or not, whether the participant lives in a rural or urban area, and the educational level of parents.

#### 2.2.2. The Multidimensional Shame Response Inventory–21 (MSRI–21)

The MSRI–21 is a recently developed instrument that measures shame by coding the respondent’s affective and behavioral responses to shame [15]. It is a multidimensional instrument that adopts a maladaptive conceptualization of shame [23]. It was first translated from English to Chinese by a research team consisting of an epidemiologist, a psychologist, and two postgraduates in epidemiology focusing on suicide prevention research, and was then back-translated to English by two bilingual experts with no prior knowledge of the original scale. By comparing the back-translated version with the original one, some items were revised and then used as the instrument. It has a total of 21 items and includes three dimensions: negative self-evaluation, fear of social consequences, and maladaptive behavior tendency. Responses are rated on a 5-point Likert scale ranging from 1 (strongly disagree) to 5 (strongly agree). Higher scores reflect higher levels of shame. Previous studies have supported the strong reliability and validity of the MSRI–21 instrument [15]. In this study, the Cronbach’s α coefficient of the scale was 0.954.

#### 2.2.3. Interpersonal Needs Questionnaire (INQ)

The INQ is a 15-item self-report measure used to assess feelings of perceived burdensomeness (6 items, INQ–PB) and thwarted belongingness (9 items, INQ–TB) [45,46]. Responses are rated on a 7-point Likert scale ranging from 1 (not at all true for me) to 7 (very true for me). Higher scores reflect higher levels of perceived burdensomeness and thwarted belongingness. The Chinese version of INQ–15 has been shown to have adequate reliability and validity in China [47]. In this study, the Cronbach’s α coefficients of thwarted belongingness and perceived burdensomeness were 0.941 and 0.797, respectively.

#### 2.2.4. Suicidal Ideation

Suicidal ideation was assessed in this study by the question “Have you ever thought about killing yourself in the past 12 months?”, which was answered “yes” or “no” by the participants. This question was also used in the US National Comorbidity Survey (NCS) [48].

#### 2.2.5. The Assessment of Anxiety and Depression

We have used Positive Affect (PA) and Somatic Anxiety (SA) from the Anxiety Depression Distress Inventory–27 (ADDI–27) which is a short version of the Mood and Anxiety Symptom Questionnaire–90 (MASQ–90) [49,50]. Either PA or SA is a self-report instrument consisting of 9 items rated on a 5-point Likert scale. Lower scores on PA reflect higher levels of depressive symptomatology. Higher scores on SA indicate increased levels of anxious symptomatology. Previous studies have supported the strong reliability and validity of the ADDI–27 instrument [49,50] and the Chinese version of ADDI–27 has been shown to have adequate reliability and validity in Chinese medical students [51]. In this study, the Cronbach’s α coefficients of PA and SA are 0.901 and 0.921 respectively.

### 2.3. Ethical Statement

The project was approved by the Institutional Review Board of the School of Public Health, Shandong University (ethical approval number is 20180401).

### 2.4. Statistical Analysis

Data were entered using EpiData 3.1 (The EpiData Association, Odense, Denmark) and analyzed by SPSS 21.0 for Windows (IBM Corp, Armonk, New York, USA). Descriptive analysis was used to present the distribution characteristics of the data. The chi-square test was conducted to assess the differences of the demographic associated with suicidal ideation, and the multiple logistic regression model was used to study the risk of suicidal ideation. The above tests were two-sided, and the significance level was set at 0.05. Hayes’ PROCESS macro [52], a regression-based analysis, was performed in mediation analysis. Based on 5000 bootstrapping samples, PROCESS was used to verify the indirect and direct effects and estimate bias-corrected confidence intervals (CIs). A direct or indirect effect was considered significant when the CI did not include a zero. A partial mediating effect was considered to exist when the direct and indirect effects were both significant. A complete mediating effect was considered to exist when the indirect effect was significant but the direct effect was not significant. Furthermore, a mediating effect did not exist when the indirect effect was not significant.

## 3. Results

### 3.1. Demographic Variables in the Sample

Table 1 presents the demographic data of the participants. Among the 2079 undergraduate students, 1041 (50.1%) were males and 1038 (49.9%) were females. The distribution of students across majors was as follows: liberal arts, 562 (27.0%); sciences, 427 (20.5%); engineering, 498 (24.0%); and medicine, 592 (28.5%). The distribution of students across grade levels was as follows: freshmen, 679 (32.7%); sophomores, 479 (23.0%); juniors, 417 (20.1%); and seniors, 504 (24.2%). More than half of the undergraduate students were from urban areas (60.2%), and 1123 (54.0%) were from one-child families.

### 3.2. Comparison of Demographic Characteristics between Undergraduates with and without Suicidal Ideation

The twelve-month prevalence of suicidal ideation among undergraduates was 8.95% (186/2079). Among the demographic variables, undergraduates with and without suicidal ideation in the past twelve months differed in major (*p* = 0.020), and there was no significant difference associated with gender, grade, family residence, ethnicity, whether they belonged to a one-child family or not, and the educational level of parents (*p* > 0.05). More details are shown in Table 1.

### 3.3. Correlation Analysis

Means, standard deviations, and correlation coefficients of shame, thwarted belongingness, perceived burdensomeness, and suicidal ideation are reflected in Table 2. The results showed that shame was positively correlated with thwarted belongingness, perceived burdensomeness, and suicidal ideation. Thwarted belongingness and perceived burdensomeness were positively correlated with suicidal ideation.

### 3.4. Risk of Suicidal Ideation among Undergraduate Students by Multivariate Logistic Regression Analysis

A multivariate logistic regression analysis was performed to identify the risk factors for suicidal ideation in the past twelve months among undergraduates (Table 3). The results showed that the individuals who were females (odds ratio OR = 1.84, *p* < 0.001), majored in liberal arts (OR = 1.74, *p* < 0.05), and had high levels of shame (OR = 1.02, *p* < 0.001), thwarted belongingness (OR = 1.02, *p* < 0.05), and perceived burdensomeness (OR = 1.07, *p* < 0.001) were statistically and positively associated with suicidal ideation. The individuals who had low levels of depression (high scores of PA) were negatively associated with suicidal ideation (OR = 0.93, *p* < 0.001).

### 3.5. Mediation Analysis

**Figure 1:**
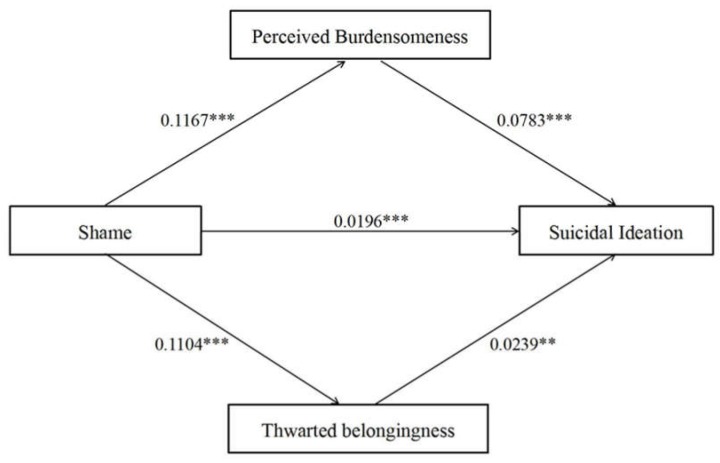
Mediating effect analysis of perceived burdensomeness/thwarted belongingness between shame and suicidal ideation; ** *p* < 0.01; *** *p* < 0.001.

Mediation analysis based on 5000 bootstrapping samples was conducted to estimate the indirect effects of shame on suicidal ideation mediated by perceived burdensomeness and thwarted belongingness. Table 4 illustrates the results of the mediation analysis. Direct effect of shame on suicidal ideation was significant (95% CI: 0.0108–0.0284), and the total indirect effect was also significant (95% CI: 0.0087–0.0151). The indirect effects of shame on suicidal ideation via the mediating effects of perceived burdensomeness and thwarted belongingness were approximately 0.0091 (95% CI: 0.0066–0.0121) and 0.0026 (95% CI: 0.0009–0.0048), respectively (Figure 1). The results revealed that there were partial mediating effects of perceived burdensomeness and thwarted belongingness between shame and suicidal ideation.

## 4. Discussion

To the authors’ knowledge, the present study is the first to apply the interpersonal theory of suicide to explore the relationship between shame and suicidal ideation in Chinese undergraduates. Our major findings were as follows: (1) suicidal ideation was prevalent among college students in China; (2) higher levels of shame, thwarted belongingness, and perceived burdensomeness were correlative factors of suicidal ideation; (3) thwarted belongingness and perceived burdensomeness mediated the association between shame and suicidal ideation.

In our study, our finding of 8.59% undergraduates with suicidal ideation in the past twelve months is lower than the prevalence found in recent studies in China (13.2%–35.3%) [49,53,54,55] and almost the same as the prevalence found in other domestic and foreign studies (2.0%–8.8%) [56,57,58]. The reasons for this phenomenon may be due to different survey regions, sampling methods, and measuring tools among college students. However, the prevalence of twelve-month suicidal ideation in many studies of the general population was lower than the prevalence in our study based on samples of undergraduate students [59,60]. The higher prevalence of suicidal ideation among college students may be due to the need to adapt to college life, self-identity issues, interpersonal relationships, and career development issues. Some of them may be confronted with negative emotions without adequate coping skills. On this basis, more attention should be given to college students with respect to suicidal ideation prevention and suicide intervention.

Suicidal ideation among undergraduates is associated with various factors. Among the demographic factors, our findings are consistent with previous studies where females were more prone to suicidal ideation [61,62,63,64,65]. However, undergraduates who major in the liberal arts are more likely to experience suicidal ideation than those in medicine. This result is inconsistent with previous studies [66], which found that there is a higher prevalence in medical students due to information overload, lack of leisure time, financial debt, being away from home, a reluctance to seek psychiatric help, and so on. The results may be due to the fact that most of the students who majored in the liberal arts in this study were females. Furthermore, some of them could have been relatively sensitive in cognition and emotion, which sometimes may lead to extreme thoughts and impulsive behaviors [23].

Consistent with the findings of previous studies, we found that shame was related to suicidal ideation in our undergraduate sample. The cross-sectional surveys among British young adults [67] and Chinese undergraduates [25] showed similar conclusions. Due to the pressure of the college entrance examination in Chinese universities, academic achievement has been prioritized over mental health in most of the elementary and secondary schools. To some extent, students were usually more proficient in achieving an excellent academic performance rather than adapting well psychologically in areas such as self-evaluation, emotional regulation, and stress management before they started their college life [68,69,70]. Furthermore, a socially defined sense of “honor” is more important than personal “dignity” in Chinese culture, so that a considerable number of students do their best to attain “honor” by living up to their family’s expectations. Meanwhile, they could likely experience shame if they failed in their academic work, interpersonal relations or other field. As a result of social and family pressure, they may be more vulnerable to psychological distress [71], and shame is an important psychological state resulting from this distress [26]. Thus, shame should be an area of concern with respect to suicidal ideation prevention in Chinese undergraduates. The particularity of undergraduates’ social position and psychological state should be further understood and investigated.

Y.J. Wong et al. [72] carried out a study on interpersonal shame among Asian Americans using the Interpersonal Shame Inventory, and they found that family shame mediated the effects of thwarted belongingness on suicide ideation. In contrast, results from this study have indicated that the association between shame and suicidal ideation is mediated by thwarted belongingness and perceived burdensomeness. This finding might be due to the cultural differences between Asian and Western countries, especially the United States, as Asians in China are more concerned about honor, i.e., gaining respect from others. When an individual fails, he or she is vulnerable to shame [73]. In addition, individuals who have experienced shame may have the desire to eliminate contact with their inner groups, families or university friends, as they fear their “failures” would be known by others. This can lead to loneliness and low levels of belongingness [74]. In more serious cases, individuals may feel that they are a burden to their families when they do not meet expectations or when they violate social norms. This occurrence may be due to the perception that the shameful event may bring disgrace upon family members, as family reputation is of crucial importance in Asian culture [75]. Shame may exert a tremendous influence on suicidal ideation in college students [76]. Therefore, this study further confirms the effects of shame on suicidal ideation based on the framework of the interpersonal theory of suicide [31,77,78].

### 4.1. Implications for Practice

Findings from this study provide further evidence for the importance of shame, thwarted belongingness, and perceived burdensomeness in the mitigation of suicidal ideation. Therefore, mental health practitioners in universities in China (e.g., college counselor) should pay attention to and routinely assess the psychological status of college students [79]. Congruent with the interpersonal theory of suicide, this study also contributes to the understanding of the mechanism between shame and suicidal ideation, which are mediated by perceived burdensomeness and thwarted belongingness. As a cognitive state of emotion, either thwarted belongingness or perceived burdensomeness could be influenced over time by interpersonal and intrapersonal factors. Therefore, we propose that suicidal ideation could be reduced effectively by changing levels of thwarted belongingness or perceived burdensomeness. For instance, life-meaning education is needed in the psychological education of college students to encourage them to establish a positive view of life and to reduce their perceptions of perceived burdensomeness. In the related life-meaning education, college students should be taught the preciousness of life, and how to deal with the stresses and frustrations in life. In addition, to reduce the negative impact of shame, the development of group activities should be vigorously promoted and made more accessible for college students. Thus, their social skills could improve, and interpersonal interactions could grow [80]. In addition, the social support of college students needs to be strengthened by establishing social networks [81], which may result in a powerful sense of belongingness to prevent suicidal ideation.

### 4.2. Limitations

First, the self-reported data were not sufficiently objective, and information bias may exist. Second, a causal relationship could not be established because of the cross-sectional design, and a longitudinal design should be attempted. Third, the MSRI–21 assesses maladaptive, affective, and behavioral responses to shame. However, the adaptive facet of shame is excluded. Fourth, we used one question to assess suicidal ideation rather than an ideation-specific rating scale to examine the prevalence of suicidal ideation. Future studies should consider measuring suicidal ideation using a standardized rating scale. Finally, although the sample size was large, the participants were selected from one university, so the findings from this sample may not be generalized to other provinces or other population groups in China.

## 5. Conclusions

The prevalence of suicidal ideation is common among Chinese undergraduates. Undergraduates in China who experienced more shame, perceived burdensomeness, and thwarted belongingness are more likely to report suicidal ideation. The interpersonal theory of suicide helps illuminate the mechanism of suicidal ideation and the relationship between shame and suicidal ideation is mediated by perceived burdensomeness and thwarted belongingness. Therefore, evaluation and effective interventions of shame, thwarted belongingness, and perceived burdensomeness would be helpful in reducing suicidal ideation among university students in China.

## Figures and Tables

**Table 1:** Description and comparison of demographic characteristics between undergraduates with and without suicidal ideation.

Variables	Total	No Suicidal Ideation	Suicidal Ideation	*χ2*	*p*
	*n* (%)	*n* (%)	*n* (%)		
Gender					
Male	1041 (50.1)	959 (92.1)	82 (7.9)	2.93	0.091
Female	1038 (49.9)	934 (90.0)	104 (10.0)	-	-
Grade					
Freshmen	679 (32.7)	623 (91.8)	56 (8.2)	1.69	0.640
Sophomores	479 (23.0)	430 (89.8)	49 (10.2)	-	-
Juniors	417 (20.1)	378 (90.6)	39 (9.4)	-	-
Seniors	504 (24.2)	462 (91.7)	42 (8.3)	-	-
Major					
Medicine	592 (28.5)	551 (93.1)	41 (6.9)	9.77	0.020
Liberal arts	562 (27.0)	497 (88.4)	65 (11.6)	-	-
Science	427 (20.5)	384 (89.9)	43 (10.1)	-	-
Engineering	498 (24.0)	461 (92.6)	37 (7.4)	-	-
Ethnicity					
Han	1912 (92.0)	1737 (90.8)	175 (9.2)	1.24	0.322
Minorities	167 (8.0)	156 (93.4)	11 (6.6)	-	-
Residence					
Rural	827 (39.8)	762 (92.1)	65 (7.9)	1.99	0.182
Urban	1252 (60.2)	1131 (90.3)	121 (9.7)	-	-
Only child or not					
Yes	1123 (54.0)	1013 (90.2)	110 (9.8)	2.16	0.144
No	956 (46.0)	880 (92.1)	76 (7.9)	-	-
Educational level of parents					
High school or lower	1290 (62.0)	1176 (91.2)	114 (8.8)	0.08	0.969
University	708 (34.1)	643 (90.8)	65 (9.2)	-	-
Master’s degree or higher	81 (3.9)	74 (91.4)	7 (8.6)	-	-

Note: *n* = frequency; *χ2* = Chi-square test value; *p* = *p-*value; in all the analysis, *p* < 0.05 was considered statistically significant.

**Table 2:** Standard deviation and correlation matrix.

Variables	M (SD)	Shame	Thwarted Belongingness	Perceived Burdensomeness	Suicidal Ideation
Shame	45.49 (±17.70)	1.00	-	-	-
Thwarted belongingness	33.51 (±11.29)	0.133 *	1.00	-	-
Perceived burdensomeness	8.26 (±5.41)	0.376 *	0.172 *	1.00	-
Suicidal ideation	0.09 (±0.29)	0.185 *	0.105 *	0.271 *	1.00

Note: M = mean; SD = standard deviation; * *p* < 0.001.

**Table 3:** Risk of suicidal ideation among undergraduate students by logistic regression.

Variables	Crude OR (95% CI)	*p*	Adjusted OR (95% CI)	*p*
Gender				
Male	1.00	-	1.00	-
Female	1.30 (0.96–1.76)	0.088	1.84 (1.25–2.70)	0.002
Grade				
Freshmen	1.00	-	1.00	-
Sophomores	1.27 (0.85–1.90)	0.248	1.10 (0.68–1.76)	0.707
Juniors	1.15 (0.75–1.76)	0.528	0.88 (0.52–1.47)	0.612
Seniors	1.01 (0.67–1.54)	0.958	0.94 (0.58–1.52)	0.811
Major				
Medicine	1.00	-	1.00	-
Liberal arts	1.76 (1.17–2.65)	0.007	1.74 (1.10–2.75)	0.017
Sciences	1.50 (0.96–2.35)	0.073	1.56 (0.95–2.54)	0.079
Engineering	1.08 (0.68–1.71)	0.748	1.28 (0.76–2.13)	0.346
Ethnicity				
Han	1.00	-	1.00	-
Minorities	0.70 (0.37–1.32)	0.268	0.63 (0.32–1.25)	0.185
Residence				
Rural	1.00	-	1.00	-
Urban	1.25 (0.92–1.72)	0.159	1.23 (0.80–1.87)	0.349
Only child or not				
Yes	1.00	-	1.00	-
No	0.80 (0.59–1.08)	0.142	0.86 (0.58–1.26)	0.429
Educational level of parents				
High school or lower	1.00	-	1.00	-
University	1.04 (0.76–1.44)	0.797	0.94 (0.63–1.40)	0.747
Master’s degree or higher	0.98 (0.44–2.17)	0.952	0.83 (0.35–1.96)	0.663
ADDI–PA	0.92 (0.91–0.94)	<0.001	0.93 (0.91–0.95)	<0.001
ADDI–SA	1.05(1.03–1.07)	<0.001	1.02 (0.99–1.04)	0.189
Shame	1.03 (1.03–1.04)	<0.001	1.02 (1.01–1.03)	<0.001
Thwarted belongingness	1.04 (1.02–1.06)	<0.001	1.02 (1.00–1.04)	0.037
Perceived burdensomeness	1.11 (1.09–1.13)	<0.001	1.08 (1.05–1.10)	<0.001

Note: OR = odds ratio; CI = confidence interval; *p* = *p*-value; crude ORs were derived from univariate nonconditional logistic regression; adjusted ORs were adjusted for all variables in the table; ADDI-PA = Positive Affect from the Anxiety Depression Distress Inventory–27; ADDI-SA = Somatic Anxiety from the Anxiety Depression Distress Inventory–27.

**Table 4:** Mediating effects of perceived burdensomeness and thwarted belongingness between shame and suicidal ideation by process.

Effect Types	Mediating Variables	Path	95% CI	Effect
Direct effect	-	Shame → Suicidal ideation	0.0108–0.0284	0.0196
Indirect effect	Perceived burdensomeness	Shame → Perceived burdensomeness → Suicidal ideation	0.0066–0.0121	0.0091
	Thwarted belongingness	Shame → Thwarted belongingness → Suicidal ideation	0.0009–0.0048	0.0026
Total indirect effect	-	-	0.0087–0.0151	0.0118
Total effect	-	-	-	0.0314
